# 3D accurate osteotomy for pediatric cubitus varus deformity using a custom-matched surgical osteotomy template combined with a reduction template via a limited lateral incision: a case report and literature review

**DOI:** 10.3389/fsurg.2026.1764504

**Published:** 2026-08-27

**Authors:** Jiang-Long Guo, Xiao Zeng, Jian-Hao Guan, Hai-Yun Chen, Kui Zhao, Mei-Ren Zhang

**Affiliations:** 1 Guangzhou University of Chinese Medicine Second Clinical College, Guangzhou, Guangdong, China; 2 Orthopedics Trauma Department, ZhuHai Hospital, Guangdong Provincial Hospital of Chinese Medicine, ZhuHai, Guangdong, China

**Keywords:** 3D accurate osteotomy, custom-matched surgical osteotomy template, limited lateral incision, pediatric cubitus varus deformity, reduction template

## Abstract

**Background:**

Cubitus varus deformity is a common complication following supracondylar fractures of the humeri in children. Due to the limited capacity of the distal humeral epiphysis to spontaneously correct existing varus deformity, the deformity typically persists into adulthood without improvement. Accurate correction of this deformity is essential to prevent late complications and significantly improve cosmetic appearance. However, achieving precise correction through a limited incision remains technically emanding.

**Case presentation:**

We present the case of a 12-year-old female who sustained a left supracondylar humeral fracture following a fall at the age of 11 years. The patient was initially treated with immobilization cast at another hospital. Four weeks after cast removal, a cubitus varus deformity was noted, which persisted for 8 months following the initial injury. The patient was subsequently to our hospital for improvement of both function and cosmesis. A three-dimensional (3D) guided accurate osteotomy was performed using a custom-made surgical template combined with a reduction template via a limited lateral incision. The operation time was 116 min, with an intraoperative blood loss of 20 mL. Postoperatively, the carrying angle and tilting angle on the affected side improved significantly, from −22.1° (varus) and 1.3° preoperatively to 12.2° (valgus) and 52.3°, respectively. Bone union was achieved at 3 months after surgery. At the 1-year follow-up, the patient had an excellent outcome with a Hospital for Special Surgery score of 98, and without recurrence of varus deformity, neurovascular injury, or wound complications.

**Conclusion:**

Accurate 3D correction of pediatric cubitus varus deformity can be successfully achieved using a custom-matched surgical osteotomy template combined with a reduction template via a limited lateral incision.

## 1 Background

Cubitus varus deformity is a common complication following supracondylar humeral fractures in children (1–4). Various methods for three-dimensional (3D) corrective osteotomy have been reported (5–8). However, these methods differ with respect to surgical incisions and internal fixation techniques, and achieving accurate osteotomy through a minimal incision remains technically challenging. Cubitus varus deformity involves three planes: varus in the coronal plane, hyperextension in the sagittal plane, and internal rotation in the horizontal plane. Herein, we introduce a 3D preoperative computer simulation-based approach using custom-printed surgical templates to achieve accurate 3D osteotomy via a limited lateral incision, and review the relevant literature.

## 2 Case presentation

A 12-year-old female presented to our hospital with concerns regarding the cosmetic appearance of her left elbow and limited elbow joint function, 8 months after sustaining a supracondylar humeral fracture. The patient had been treated initially with an immobilization cast for 4 weeks, after which the cubitus varus deformity persisted, becoming noticeable to her parents. Preoperative photographs, along with anteroposterior (AP) and lateral x-rays confirmed cubitus varus deformity of the left elbow. The preoperative carrying angles of the normal and affected sides were 15.3° and −22.1° (varus), respectively, and the tilting angles (TA) were 52.1° and 1.3°, respectively. The elbow's range of motion had returned to within normal limits (0°–120°), but showed a 30° reduction compared with the unaffected side. Hyperextension of the elbow and internal rotation of the shoulder were normal. The patient underwent 3D accurate osteotomy using a custom-made 3D printed surgical template combined with a reduction template via a limited lateral incision. Informed consent was obtained from the patient and her parents prior to the publication of this case report.

## 3 Surgery treatment

### 3.1 Step 1: 3D evaluation of cubitus varus deformity and planning of the 3D corrective osteotomy

**Figure 1:**
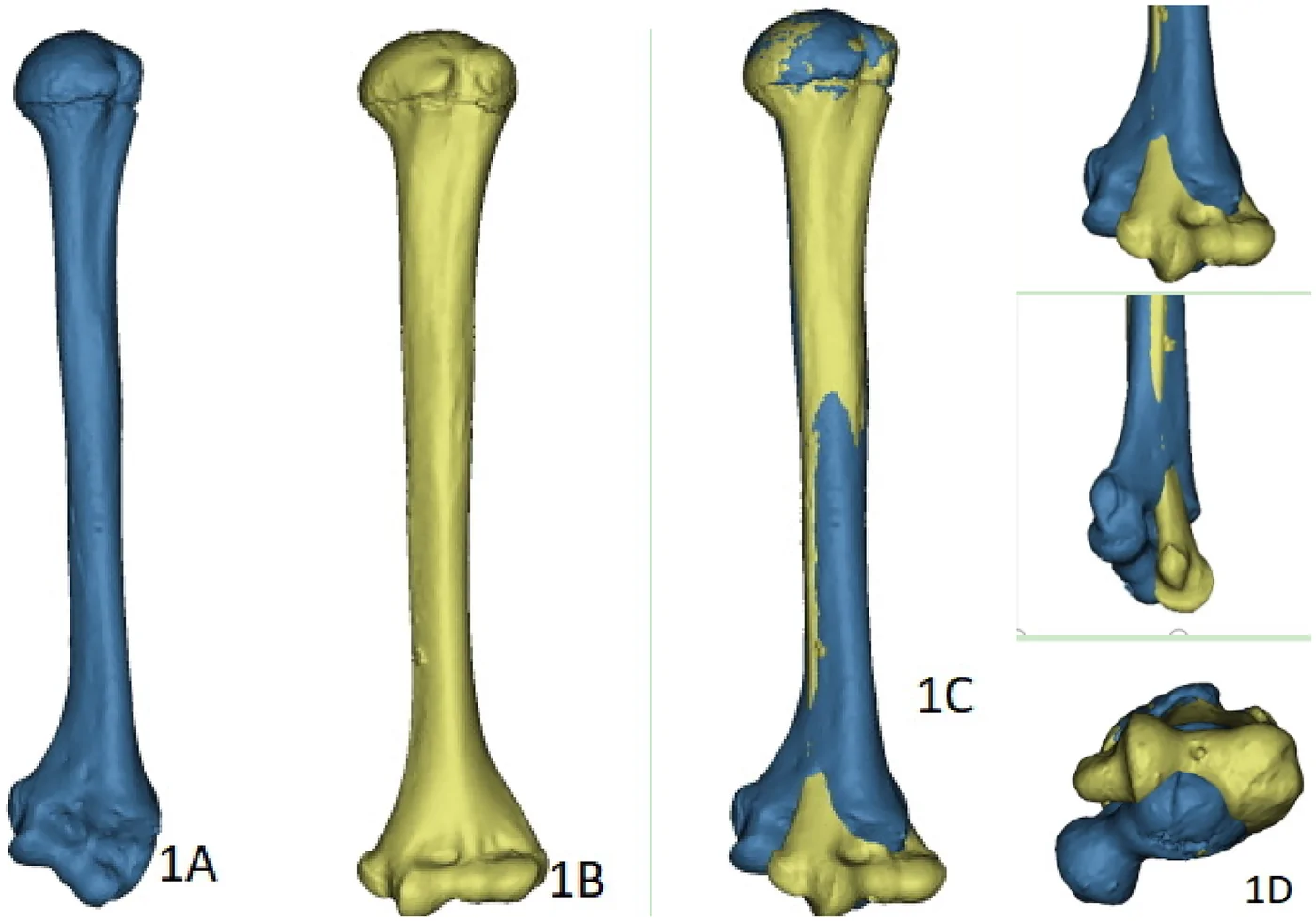
Three-dimensional deformity evaluation. **(A)** Affected humeri (dark blue). **(B)** Mirror image of contralateral normal humeri (yellow). **(C)** Proximal superimposition of affected and mirror images. **(D)** Quantified 3D deformity (varus, extension, internal rotation).

**Figure 2:**
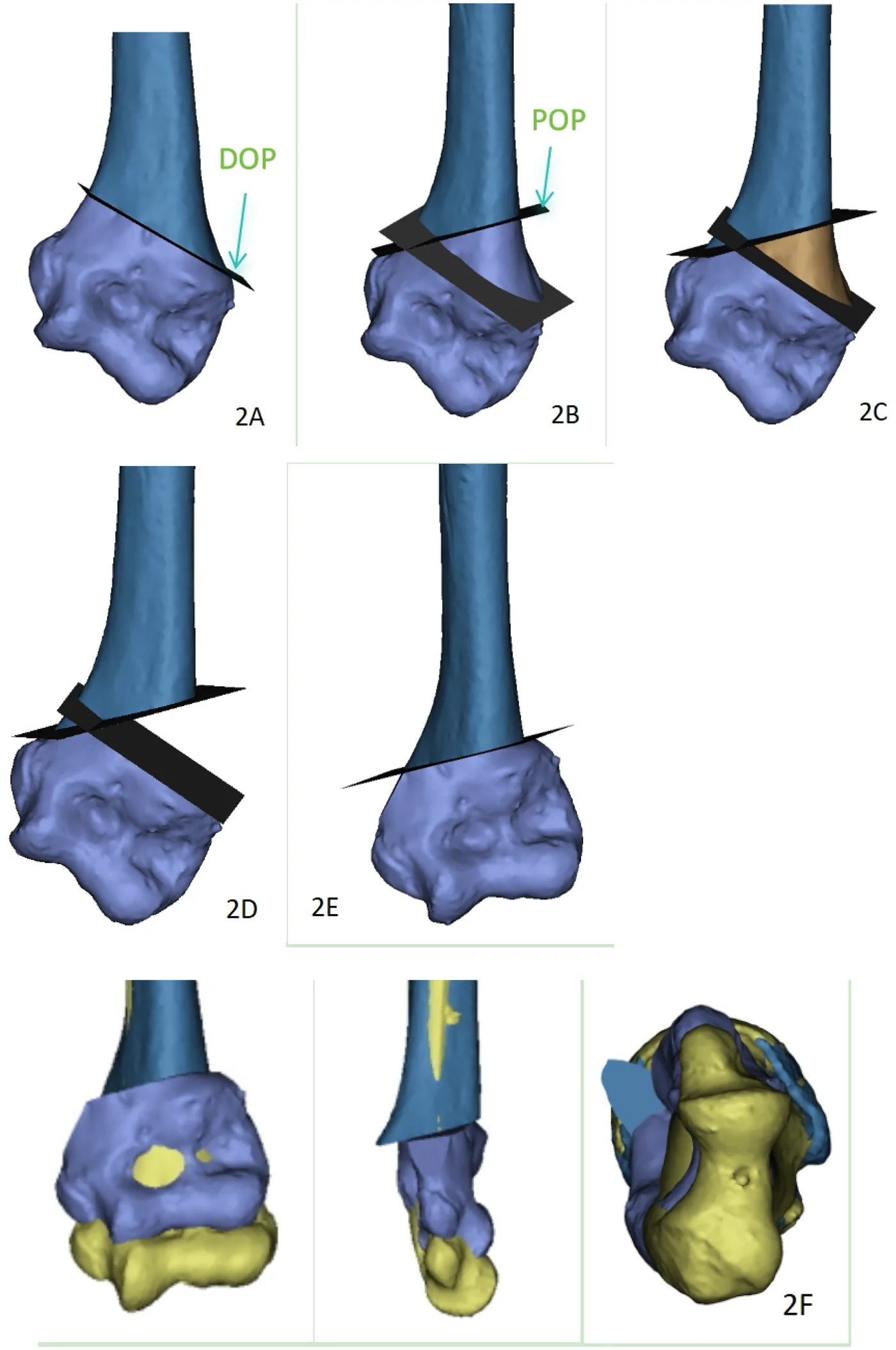
Planning of 3D corrective osteotomy. **(A)** Distal osteotomy plane (DOP) set just proximal to the olecranon fossa. **(B)** Proximal osteotomy plane (POP) determined by superimposing the mirror image of the contralateral distal humeri. **(C,D)** Wedge-shaped segment (orange) removed. **(E)** Simulation of completed 3D correction. **(F)** Post-correction verification on computer monitor.

Both humeri were scanned with the forearms in maximum supination using a CT scanner (Computerized Tomography General Electric LightSpeed-640, GE, Milwaukee, WI) with a low-radiation-dose technique. 3D bone-surface models of both humeri were created from digital data and evaluated the deformity three-dimensionally by comparing the affected humeri to a mirror image of the contralateral normal humeri using specialized 3D software (Magics RP; Materialise, Leuven, Belgium) (Figures 1A,B). The affected humeri was superimposed onto the mirror image of the corresponding part of the contralateral normal side (proximal registration) by translating and rotating the two humeral models until they matched precisely (Figure 1C). The computer software automatically calculated the 3D deformity based on the transformation data from distal and proximal registration (Figure 1D). A simulated 3D corrective osteotomy was then performed based on the deformity evaluation data. With the proximal portion of the affected humeri aligned to the mirror image of the contralateral normal side, we placed an osteotomy plane approximately parallel to the distal articular surface and just proximal (0–1 cm) to the olecranon fossa, this was designated as the distal osteotomy plane (DOP) (Figure 2A). Next, the distal humeri from the normal side was precisely superimposed onto the distal part of the affected side by translation and rotation, and the proximal osteotomy plane (POP) was established by shifting the distal osteotomy plane according to the magnitude of correction required (Figure 2B). The wedge-shaped segment defined by the DOP and POP was then removed from the affected humeri using the software's editing function (Figures 2C,D). The 3D correction simulation was completed by moving the distal humeral segment to align with the proximal segment (Figure 2E). Finally, it was verified on the computer monitor that the overall appearance of the extremity had been correctly restored (Figure 2F).

### 3.2 Step 2: design of patient-specific surgical guides (including surgical osteotomy and reduction templates)

#### 3.2.1 Design of the custom-made surgical reduction template

**Figure 3:**
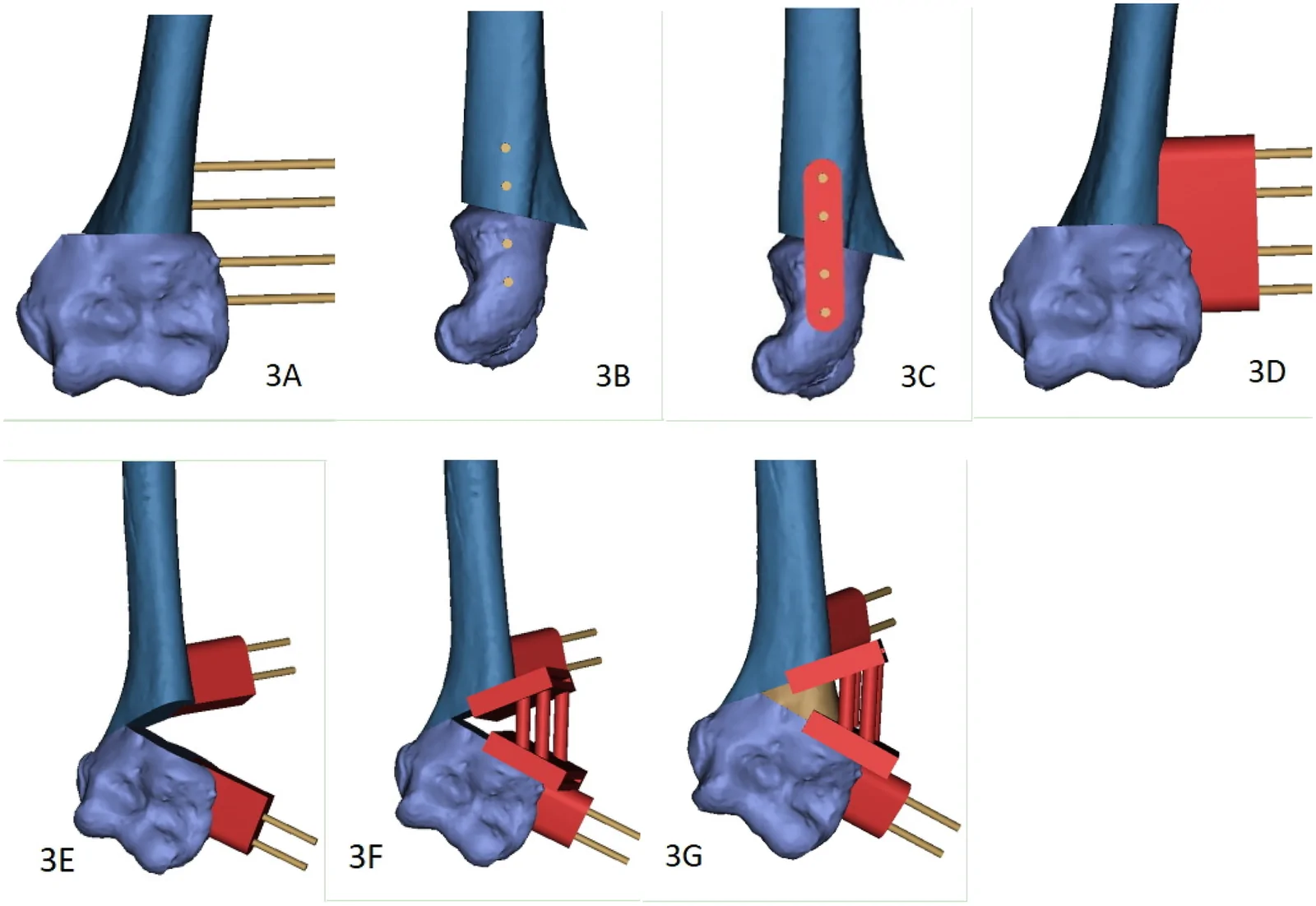
Design of custom-made surgical templates. **(A,B)** Placement of four parallel guide pins perpendicular to humeral axis. **(C,D)** Custom-made reduction template designed to match lateral distal humeri. **(E)** Reduction template separated at osteotomy plane. **(F)** Two osteotomy slits designed along POP and DOP, connected to reduction template parts. **(G)** Completed osteotomy template.

The wedge-shaped segment defined by the DOP and POP was removed from the affected humerus. The simulation 3D correction was completed by aligning the distal humeral segment with the proximal humeral segment. The reduction guide was shaped to precisely fit the posterolateral surface of the distal humeri after the 3D correction had simulated. Four guide pins (2.0 mm in diameter each) were placed parallel to one another, spaced at intervals of 8 mm along the midline of the lateral distal humeri, perpendicular to the humeral axis (Figure 3A). The midpoint between the second and third guide pins was positioned directly on the osteotomy surface (Figure 3B). Using SolidWorks software, the custom-made surgical reduction guide was then designed based on these four guide pins and their exact positions, ensuring it precisely matched the lateral side of the distal humeri (Figures 3C,D). This guide was designed to facilitate accurate reduction and temporary fixation following osteotomy.

#### 3.2.2 Design of the custom-made surgical osteotomy template

**Figure 4:**
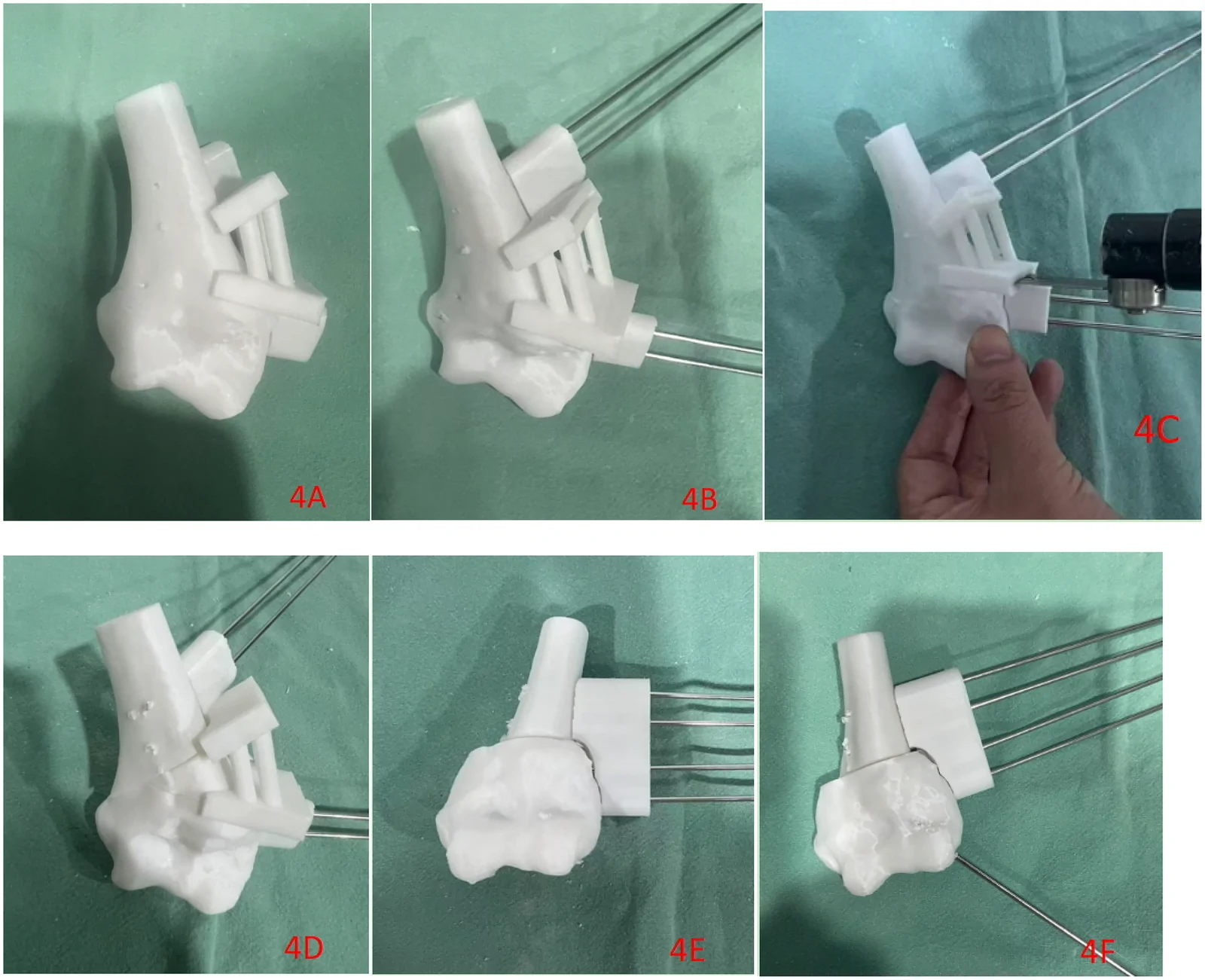
Preoperative simulation of 3D corrective osteotomy using custom-made templates on a 3D-printed model. **(A,B)** Osteotomy template positioned and fixed with pins. **(C,D)** Osteotomy performed through slits. **(E)** Bone wedge removed and K-wires aligned in parallel. **(F)** Reduction template maintaining parallel K-wire position with additional temporary fixation.

The custom-made surgical reduction guide was divided into upper and lower parts at the osteotomy surface using the software, and the distal part of the affected humerus was returned to its original uncorrected position (Figure 3E). Two osteotomy slits were designed along the POP and the DOP, each connected to the upper and lower sections of the surgical reduction guide by a connecting rod, thereby enhancing the stability of the surgical osteotomy guide (Figure 3F). The osteotomy guide was finalized by precisely matching it to the lateral side of the distal humerus using SolidWorks software (Figure 3G). A preoperative simulation of the 3D corrective osteotomy was conducted using the custom-made osteotomy template combined with the reduction template on a 3D-printed model (Figures 4A–F).

### 3.3 Step 3: surgical technique

**Figure 5:**
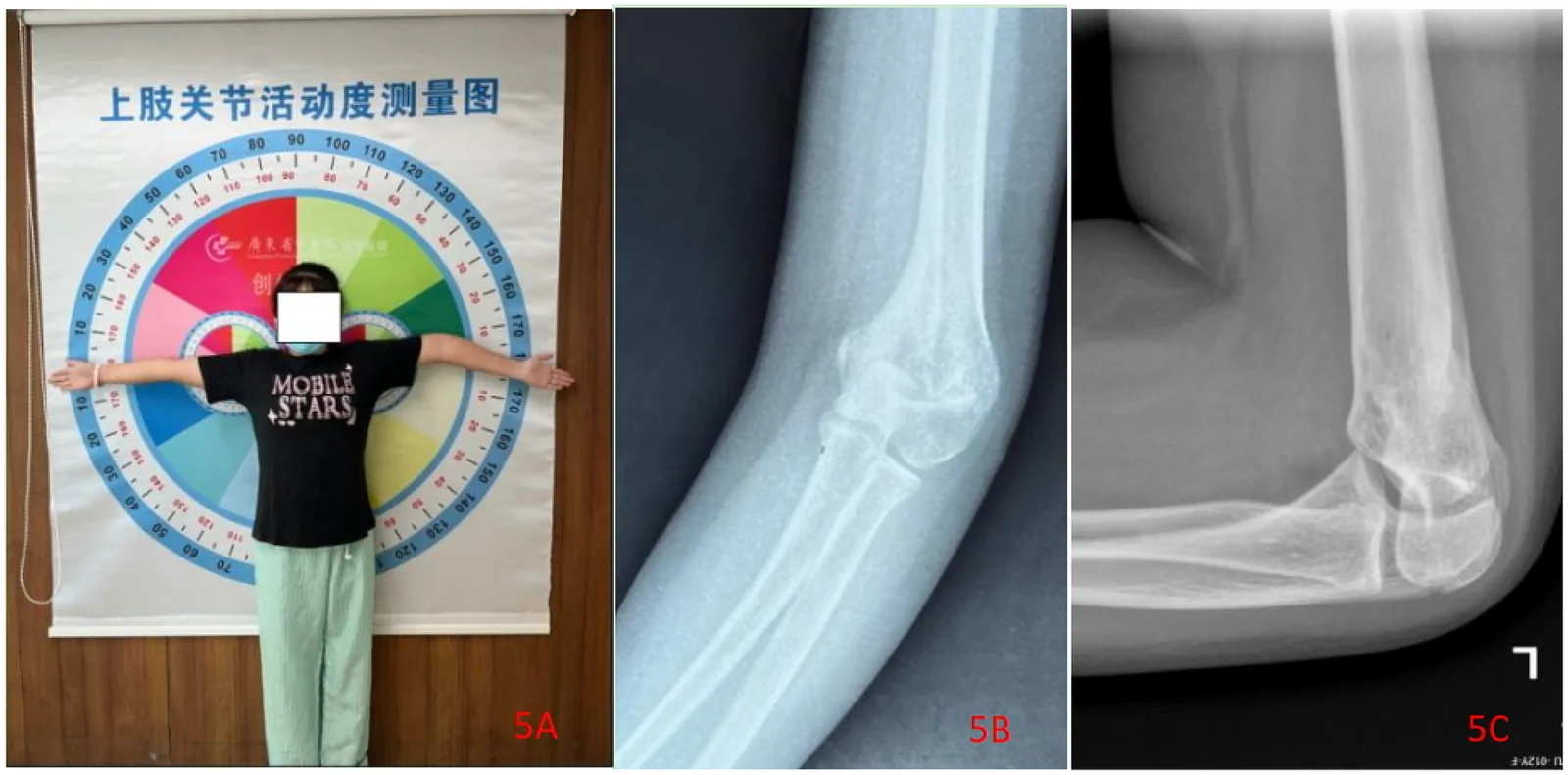
Preoperative presentation of left cubitus varus deformity in a 12-year-old girl. **(A–C)** Clinical photographs and radiographs showing varus deformity.

**Figure 6:**
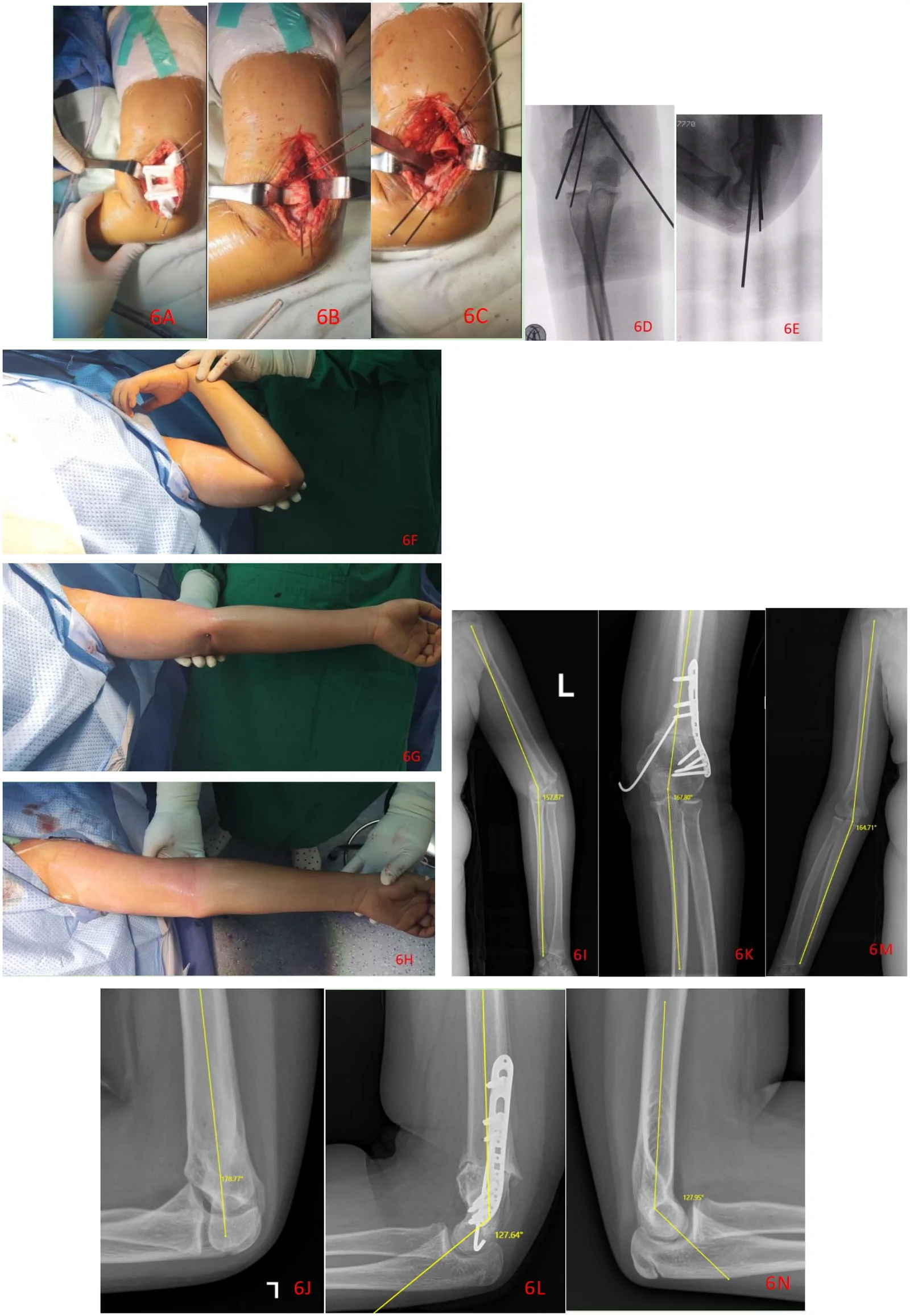
Intraoperative use of custom-made templates and postoperative radiographs. **(A)** Osteotomy template positioned and fixed with K-wires. **(B)** Osteotomy performed through slits. **(C)** Wedge-shaped bone removed. **(D,E)** Temporary fixation with reduction template and additional K-wires. **(F–H)** Normal passive range of motion immediately postoperatively. **(I–N)** Preoperative and postoperative AP and lateral x-rays showing accurate correction.

The patient underwent osteotomy for cubitus varus deformity (Figures 5A–C) using the custom-made surgical template combined with the reduction template via a limited lateral incision under general anesthesia and was positioned supine. A tourniquet was routinely applied and inflated to 200 mmHg. A limited lateral incision extending from 6 cm proximal to 2 cm distal to the elbow crease was made without exposure of the radial nerve. Four 2.0 mm Kirschner wires were inserted through guide pipes on the custom-made surgical osteotomy template after all edges of the guide were confirmed to be in complete contact with the lateral surface of the distal humerus (Figure 6A). Osteotomy was performed using a saw guided by the cutting slits in the osteotomy template (Figure 6B). The wedge-shaped bone segment was then removed (Figure 6C). The planned correction was achieved by aligning the four Kirschner wires parallel to each other and securing them temporarily with the reduction template, ensuring complete contact with the bone surface. Subsequently, an additional three Kirschner wires were inserted for temporary fixation (Figures 6D,E). Internal fixation was performed using a lateral Locking Compression Plate (LCP) after removing the reduction template; lateral Kirschner wires were then removed while medial Kirschner wires were retained percutaneously. Alignment was confirmed intraoperatively using C-arm fluoroscopy. The passive range of motion immediately after surgery was normal, and the cubitus varus deformity was corrected through an 8 cm lateral incision (Figures 6F–H). The periosteum was sutured, and the skin was closed without drainage. Total operative time was 116 min, with intraoperative blood loss of 20 mL.

## 4 Results

AP and lateral x-rays demonstrated accurate correction of the cubitus varus deformity of the left elbow. The carrying angle and tilting angle on the affected side significantly improved from −22.1° (varus) (Figure 6I) and 1.3° (Figure 6J) preoperatively, to 12.2° (valgus) (Figure 6K) and 52.3° (Figure 6L), respectively, postoperatively, closely matching the contralateral normal side (Figures 6M,N). Active and passive range-of-motion exercises were permitted on the day following surgery without splint protection.

**Figure 7:**
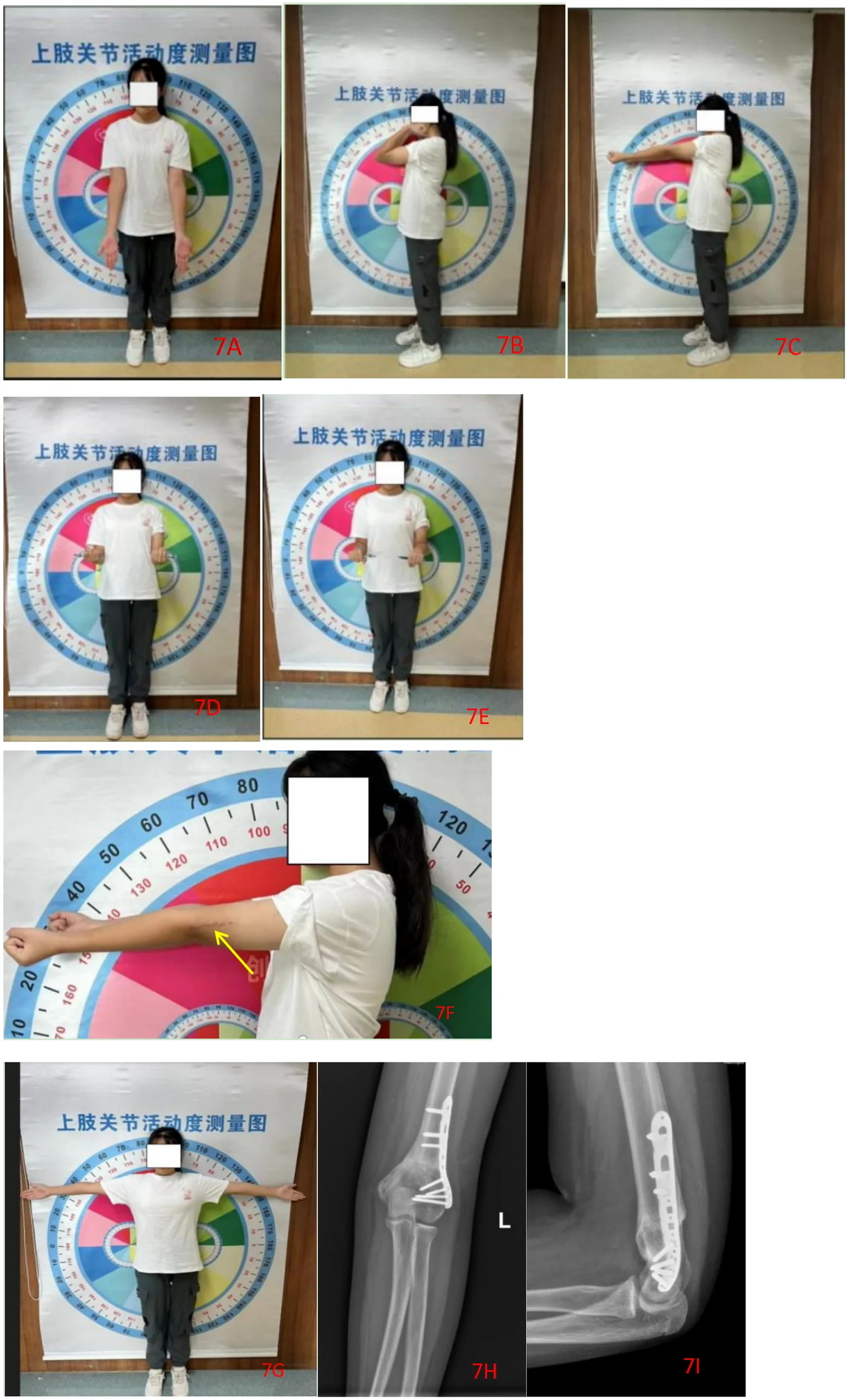
One-year postoperative follow-up. **(A–E)** Clinical examination showing nearly identical bilateral elbow alignment and full range of motion. **(F)** Well-healed 8 cm lateral incision. **(G–I)** Postoperative photographs and radiographs confirming accurate correction comparable to the contralateral normal sid.

Medial Kirschner wires were removed 2 months postoperatively, and complete bone union was achieved 3 months after surgery. The patient underwent reoperation for asymptomatic hardware removal and had a full range of motion (Figures 7A–E), good strength, and returned to her previous level of sporting activity. The patient had an excellent outcome according to the Hospital for Special Surgery score, without recurrence of varus deformity, neurovascular injury, or wound complications (Figure 7F). Photographs and AP and lateral x-rays confirmed accurate correction of the cubitus varus deformity of the left elbow at the 1-year follow-up (Figures 7G–I). A detailed clinical timeline is presented in Table 1.

**Table 1:** Clinical timeline from initial injury to final follow-up.

Time point	Event
Age 11	Fall injury, left supracondylar humeral fracture
	Cast immobilization for 4 weeks at another hospital
	Cubitus varus deformity noted after cast removal
8 Months post-injury (age 12)	Presented to our hospital (age 12 years)
	Clinical examination and radiographs
	Bilateral CT scan and 3D deformity evaluation
	3D planning and design of custom templates
	Surgery: 3D-guided osteotomy with dual templates
	Limited lateral incision (8 cm), operative time 116 min, blood loss 20 mL
Post-op day 1	Active and passive range-of-motion exercises started
2 Months	Medial Kirschner wires removed
3 Months	Bone union achieved
1 Year	Final follow-up: HSS score 98, full range of motion
	No deformity recurrence, no neurovascular injury
	Well-healed scar

## 5 Discussion

Cbitus varus deformity is the most common complication of distal humeral fractures in adolescents (9). Several surgical techniques have been reported to correct cubitus varus deformity, including lateral closing-wedge osteotomy, French osteotomy, pentalateral osteotomy, and 3D printing-assisted osteotomy (5, 10). However, conventional techniques often rely on intraoperative fluoroscopy and surgeon experience to determine the correction angle, which may lead to under-correction or residual deformity.

Recent studies have demonstrated that cubitus varus deformity is a 3D deformity involving not only varus angulation but also extension and internal rotation of the distal humeral segment (11–13). Accordingly, 3D correction has increasingly become the focus of surgical treatment, aiming for complete improvement in both appearance and function. Multiple studies have demonstrated the advantages of three-dimensional correction over traditional 2D approaches. Omori et al. (14) quantitatively analyzed the accuracy of 3D corrective osteotomy using custom-made surgical guides and found that computer simulation-based correction significantly improved postoperative alignment with minimal deviation from the preoperative plan, thereby enhancing reproducibility and reducing residual deformity. Takeyasu et al. (15, 16) further validated the efficacy of a custom-designed surgical device in achieving precise multiplanar correction, emphasizing that 3D planning allows for individualized osteotomy design and reliable execution even in complex deformities. Additionally, Tanwar et al. (17) introduced a triple modified French osteotomy technique, highlighting that a comprehensive understanding of the three-dimensional nature of cubitus varus deformity is essential for optimizing both angular and rotational correction.

Despite the importance of 3D correction, accurately achieving the desired correction of each dimension during surgery remains technically challenging. Surgeons often require repeated adjustments or relying solely on general appearance for guidance, increasing the risk of residual deformity (9). Recent advances in computer simulation technology, patient-matched surgical guides, and 3D printing have addressed these challenges, enabling accurate, simple, and safe 3D correction (9, 16, 18–20, 25). Several authors have reported excellent outcomes with improved accuracy in osteotomy using 3D technology, and they have recommended this approach as an ideal treatment for cubitus varus deformity (9, 14, 16, 20, 21).

However, compared with conventional procedures, corrective osteotomy using 3D surgical templates may require a larger surgical field or incision position the template. For example, Sri-Utenchai (22) used a standard posterior paratricipital approach with a 20-cm posterior midline incision, which provides excellent exposure of the distal humeri but inevitably leads to more soft tissue dissection, prolonged postoperative pain, and a less cosmetic scar. Similarly, Zhang et al. (9) reported using a double-incision approach with routine medial and lateral incisions, each approximately 5–6 cm in length, to allow simultaneous placement of the osteotomy guide and internal fixation devices. While these approaches ensure accurate correction, they do so at the cost of greater soft tissue exposure, increased surgical trauma, and potentially longer recovery times.

In contrast, we achieved accurate 3D corrective osteotomy of cubitus varus deformity using a single limited lateral incision of only 8 cm. This incision length was sufficient to fully accommodate our custom-matched surgical osteotomy template and reduction template, despite the fact that our patient-matched surgical osteotomy guide was relatively larger than those reported in previous studies (9, 22–24). The primary innovation lies in the dual-template system, which integrates both osteotomy and reduction guides. Unlike prior studies that employed an osteotomy guide alone (9, 23), our reduction template actively facilitates the reduction step by maintaining the precise parallel alignment of four Kirschner wires, thereby eliminating the need for repeated intraoperative fluoroscopic adjustments or trial reductions. Consequently, the reduction template ensures that the distal humeral fragment is positioned exactly as planned preoperatively, independent of the surgeon's visual estimation or tactile feedback. This parallel K-wire alignment technique significantly reduces operative time and radiation exposure. In our case, total operative time was 116 min with minimal fluoroscopy use, contrasting sharply with conventional freehand techniques that often require multiple fluoroscopic checks (14, 16).

Importantly, our approach avoids enlarging the surgical incision. Although a relatively large osteotomy guide is necessary to ensure stable contact with the lateral humeral surface, the 8 cm incision remained adequate because the guide and template were inserted sequentially rather than simultaneously. The reduction template, which features a low-profile design, is placed only after the osteotomy is completed and the bone wedge is removed, fitting easily through the same incision. This stands in contrast to double-incision approaches that require separate exposures for guide placement and internal fixation (9), or the extensive posterior approach that exposes the entire distal humerus (22). Finally, our method is fully compatible with early postoperative rehabilitation. The combination of temporary medial K-wires and lateral locking compression plate (LCP) fixation provided sufficient rotational and bending stability to permit active range-of-motion exercises on the first postoperative day without splint immobilization. Early mobilization is critical in pediatric patients to prevent joint stiffness and accelerate functional recovery, whereas some previous guide-assisted techniques necessitated longer immobilization periods due to less stable fixation constructs (23, 24).

In summary, our model offers a favorable balance between surgical accuracy and minimal invasiveness. The patient achieved an excellent outcome, as evidenced by a Hospital for Special Surgery score of 98. The carrying angle improved from −22.1° (varus) preoperatively to 12.2° (valgus) postoperatively, while the tilting angle improved from 1.3° to 52.3°, closely approximating the contralateral normal side (15.3° valgus and 52.1°). There was no recurrence of the varus deformity, no loss of correction, and no wound-related complications. Bone union was achieved at approximately 3 months postoperatively. This case demonstrates that accurate 3D correction of pediatric cubitus varus deformity can be achieved through a limited lateral incision using a custom-matched osteotomy template and reduction template, with satisfactory cosmetic and functional outcomes and stable fixation.

Several limitations of our approach warrant discussion. First, the use of custom-matched surgical templates and 3D planning software involves additional costs, including preoperative CT scanning, software licensing (e.g., Magics RP, SolidWorks), and 3D printing of the osteotomy and reduction templates. These expenses may limit the widespread applicability of this technique in resource-limited settings, although this barrier is likely to diminish as 3D printing technology becomes more accessible. Second, our protocol requires bilateral humeral CT scanning to obtain a mirror image of the contralateral normal side for deformity analysis, exposing the patient to additional radiation on the unaffected side. Nevertheless, we employed a low-radiation-dose technique for all scans to minimize exposure, and alternative approaches using single-side CT combined with virtual mirroring or statistical shape models may further reduce radiation burden in the future. Clinicians adopting this technique should weigh the benefits of accurate 3D correction against the incremental cost and radiation exposure, and selectively apply it in complex deformities where conventional methods are likely to yield suboptimal results.

## 6 Conclusion

Accurate 3D correction of pediatric cubitus varus deformity can be effectively achieved using a custom-matched surgical osteotomy template combined with a reduction template through a limited lateral incision.

## Data Availability

The raw data supporting the conclusions of this article will be made available by the authors, without undue reservation.
